# Characterization of Melanoma in Hungary Based on a Retrospective Single-Center Study Between 2001 and 2018

**DOI:** 10.3390/cancers17132171

**Published:** 2025-06-27

**Authors:** Renáta Gubán, Petra Parrag, Mihály Tamás Kispál, Kata Czirbesz, Tímea Danyi, István Kenessey, Gabriella Liszkay

**Affiliations:** 1 Department of Dermato-Oncology, National Institute of Oncology and National Tumor Laboratory, 1122 Budapest, Hungary; guban.renata@stud.semmelweis.hu (R.G.); kispal.mihaly@oncol.hu (M.T.K.); czirbesz.kata@oncol.hu (K.C.); danyi.timea@oncol.hu (T.D.); 2 National Cancer Registry, National Institute of Oncology and National Tumor Laboratory, 1122 Budapest, Hungary; parrag.petra@phd.semmelweis.hu; 3 Doctoral College, Semmelweis University, 1085 Budapest, Hungary; 4 Department of Pathology, Forensic and Insurance Medicine, Semmelweis University, 1091 Budapest, Hungary; 5 Central-Eastern European Academy of Oncology, 1125 Budapest, Hungary

**Keywords:** melanoma, epidemiology, clinical sample, prognosis, survival

## Abstract

**Background/Objectives:** Over the past few decades, the incidence of melanoma has been steadily rising. In Hungary, the National Institute of Oncology serves as the national center for the diagnosis and treatment of malignancies, including melanoma. This study aims to analyze our patients’ data, providing a comprehensive characterization of melanoma across the country. **Methods:** We systematically analyzed the clinico-pathological data of melanoma patients treated in the Department of Oncodermatology between 2001 and 2018. Prognostic parameters were collected from the Hospital Information System of the Institute, including gender, age, tumor location, exulceration of primary lesion, Clark level, and Breslow thickness. In addition, survival analysis was also performed. **Results:** A total of 6267 melanoma patients were included in the study, with a slight predominance of female cases. The most common tumor location was the trunk. Female patients and younger age groups were more likely to present with lower Breslow thickness, while tumors in the head region were associated with increased thickness. Over the study period, the annual median Breslow thickness showed a significant decline. Female gender, younger age, and lower Breslow thickness was correlated with improved overall survival. Over time, the studied patient population exhibited better outcomes. **Conclusions:** Despite the rising incidence of melanoma in Hungary, the annual median Breslow thickness in our studied patient group showed a decreasing trend, accompanied by improved mortality outcomes. This highlights the critical role of secondary prevention measures and their effectiveness, as well as the impact of novel therapeutic advancements.

## Simple Summary

In parallel with international data, in Hungary the incidence of melanoma increased steadily. Based on the melanoma patients treated in the National Institute of Oncology between 2001 and 2018, our study aimed to assess trends in the change of the most important prognostic factors and provide survival analysis. Our material included 6267 melanoma patients, which revealed a significant decline of annual median Breslow thickness over the study period. Age, gender, localization of the primary tumor and Breslow thickness showed association with outcome. The observed improvement of overall survival may be explained by the efficacy of secondary prevention as well as the introduction of novel therapeutic approaches.

## 1. Introduction

Melanoma, a tumor originating in approximately 90% of the cases from the melanocytes of the skin, is one of the most aggressive forms of cancer. Its rising incidence worldwide is posing considerable burdens on society and public health [1]. Despite accounting for only about 1% of all skin cancers, melanoma is responsible for more than 75% of skin cancer-related deaths, primarily due to late detection and rapid progression [2]. Among all malignancies, the incidence of melanoma showed an extreme increase over the past decades by 41%, rising globally from 230,000 cases in 2012 to 325,000 in 2020 [3]. According to GLOBOCAN’s 2022 estimates, melanoma is currently the 17th most common cancer worldwide, with over 300,000 new cases annually [4]. By 2040, it is projected to become the second most common cancer after breast cancer [5].

However, melanoma has a relatively favorable prognosis in high-income countries, where the overall 5-year survival rate exceeds 90% [2]. This is partly because most newly diagnosed melanomas are detected in localized form with a potentially good surgical response. Before 2011, early detection was crucial due to the lack of effective therapies for advanced melanoma. The introduction of targeted therapy and immunotherapy has since transformed the prognosis, making advanced melanoma patients potentially eligible for curative treatment.

The most significant risk factor for melanoma is exposure to UV radiation, including frequent sunburns—particularly in childhood—and the use of sun beds [6,7,8]. Unsurprisingly, Australia has the highest incidence of melanoma, due to its high UV exposure and a predominantly fair-skinned population [4]. According to GLOBOCAN estimates, in 2022, the melanoma incidence rate in Australia was 64.5 cases per 100,000 people, compared to 19.6 per 100,000 in Europe [4]. The rising popularity of solariums and suntanning throughout the 20th century, especially among the white population—young women in particular—has further contributed to the increasing melanoma incidence.

Although Hungary is among the countries that show the highest cancer incidence and mortality rates, melanoma occurrence in Hungary is considered moderate compared to other European nations [9]. In addition, Liszkay et al. identified a significant trend shift, with melanoma incidence and mortality decreasing in the past few years [10]. The latter phenomenon may be attributed to improved early detection and the introduction of novel therapeutic options for advanced-stage melanoma.

In Hungary, the National Institute of Oncology (NIO) is a comprehensive cancer center and leading institute for diagnosing and treating malignancies, managing approximately 20% of all oncology patients. It provides comprehensive care with state-of-the-art diagnostic techniques and advanced therapeutic options in surgery, radiotherapy and medical treatment. This study aimed to evaluate prognostic factors in melanoma patients treated at the NIO between 2001 and 2018.

## 2. Materials and Methods

### 2.1. Patient Selection and Data Collection

A total of 6267 melanoma patients were documented in the clinical database of the Department of Dermato-Oncology at the National Institute of Oncology, Hungary, between 1 January 2001 and 31 December 2018. The following prognostic parameters were collected from the hospital information system of the National Institute of Oncology: gender, age, localization (head and neck, ear, uveal, mucosa, trunk, upper and lower extremity, genital and anal regions), presence of exulceration, Clark level, and Breslow thickness of the melanoma (which measures the tumor’s depth from the skin surface to its deepest point). We also recorded the date of diagnosis and, if applicable, the date and cause of death. Vital information was recorded in the hospital information system of the Institute. Where necessary, this parameter was supplemented by registered data from the Hungarian National Cancer Registry (HNCR), which regularly receives vital statuses of the patients from the National Health Fund. Based on the death certificates adjusted by the Hungarian Central Statistical Office, HNCR’s database also contained vital status and cause of death data of the registered patients.

### 2.2. Statistical Analysis

The normality of numeric variables was assessed using the Shapiro–Wilk test. For descriptive statistics, median values, confidence intervals, and interquartile ranges (IQR) were reported for samples with non-normal distribution. Group comparisons were conducted using the non-parametric Mann–Whitney test or the Kruskal–Wallis test with post hoc analysis. Correlations were determined using Spearman’s rank-order test. The magnitude of the relationship was tested using a simple linear regression model. Overall survival analyses were performed using the Kaplan–Meier method. Overall survival intervals were determined as the time from initial diagnosis to death from any cause. The comparison between survival functions for different strata was assessed with the log-rank statistics. The effect of different confounding factors on overall survival was analyzed by the Cox proportional hazard model. A *p*-value of less than 0.05 was considered statistically significant. Statistical analyses were performed using Statistica v13.4 (TIBCO Software, Palo Alto, CA, USA), PAST version 1.86b (copyrighted by Hammer and Harper) and R v3.6.3 (The R Project for Statistical Computing, Vienna, Austria).

## 3. Results

Of the 6267 patients, 2970 were male (47.4%) and 3297 were female (52.6%). The median age at diagnosis was 60.5 years for males (range: 7–94) and 55 years for females (range: 10–93) (Table 1). Most melanomas were discovered at Clark level IV (1901 cases; 30.3%) and Clark level III (1829 cases; 29.2%), with a median Breslow thickness of 1.22 mm (range: 0.03–50 mm). The most common histology subtype was superficial spreading melanoma (35.4%); however, this parameter was not available in 44.7% of all cases, which limited the feasibility of further analysis. Tumor ulceration was present in 603 cases (9.6%), absent in 1183 cases (18.8%), and unreported in the remaining 4481 cases (71.5%). The most frequent tumor location was the trunk (2776 cases, 45.3%), followed by the lower extremities (1320 cases, 21.5%), upper extremities (1061 cases, 17.3%), and the head and neck region (690 cases, 11.3%).

A comparison of the parameters between female and male patients revealed that female patients were diagnosed at a younger age (Table 2). The trunk was the most common tumor localization for both females (1202 cases; 37.1%) and males (1574 cases; 54.4%), whereas lower limb melanomas were more common in females (971 cases; 30.0%) than in males (348 cases; 12.2%). Furthermore, despite the small number of cases, melanoma of the genital region was more prevalent in females. While data limitations restricted further evaluation, exulceration appeared to be more common in male patients than in females.

**Figure 1:**
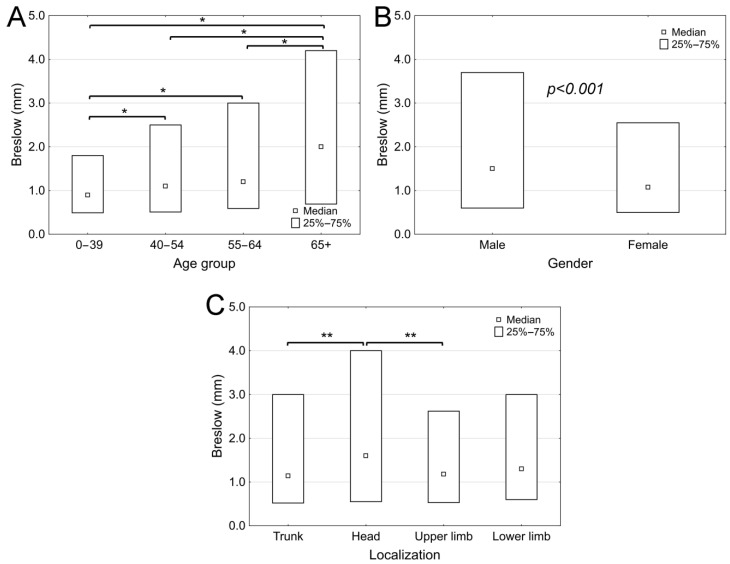
Breslow thickness of melanoma patients at the National Institute of Oncology between 2001 and 2018, categorized by age group (**A**), gender (**B**), and localization (**C**). (*: *p* < 0.01; **: *p* < 0.001).

The distribution of the studied population revealed that melanoma was discovered with a higher Breslow value in older patients (age group 0–39: median 0.895 mm (range: 0.04–40 mm); age group 40–54: median 1.1 mm (range: 0.1–41.7 mm); age group 55–64: median 1.2 mm (range: 0.05–42 mm); age group 65+: median 2 mm (range: 0.03–50 mm); *p* < 0.0001) (Figure 1A). When comparing the Breslow thickness at the date of diagnosis between the two genders, a significant difference was observed (*p* < 0.0001) in favor of women, with female patients typically diagnosed with a lower Breslow thickness (median: 1.075 mm; range: 0.03–50 mm) compared to male patients (median: 1.5 mm; range: 0.05–45 mm) (Figure 1B). Melanomas located on the head had significantly greater Breslow thickness (median: 1.6 mm; minimum: 0.1 mm; maximum: 32 mm) compared to other studied locations, such as the trunk (median: 1.14 mm; minimum: 0.05 mm; maximum: 45 mm), the upper extremities (median: 1.18 mm; minimum: 0.03 mm; maximum: 50 mm), or the lower extremities (median: 1.3 mm; minimum: 0.04 mm; maximum: 40 mm), as shown by the higher Breslow thickness (*p* = 0.003) (Figure 1C).

**Figure 2:**
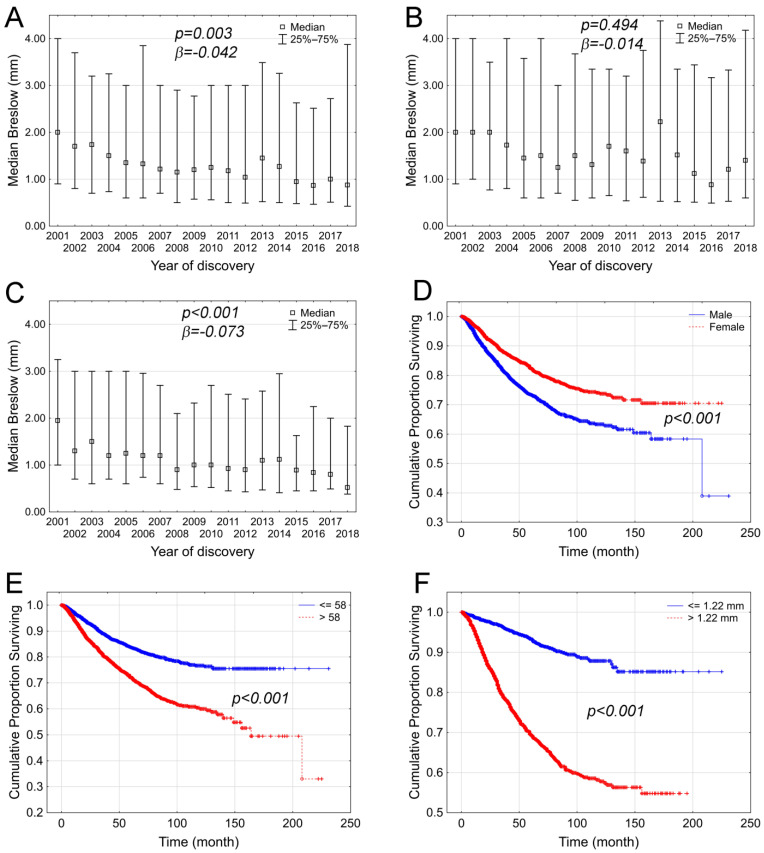
Breslow thickness and selected prognostic factors affecting the survival of melanoma patients at the National Institute of Oncology between 2001 and 2018. During the studied period, the median Breslow thickness showed a significant decrease in the total population (**A**), male patients (**B**), and female patients (**C**). Significant differences in overall survival were observed by gender (**D**), median age (**E**), and Breslow thickness (**F**).

During the study period, the annual median significantly correlated with Breslow thickness among the total patient group (*p* < 0.001; R = −0.801; Figure 2A) as well as among males (*p* = 0.016; R = −0.530, Figure 2B) and females (*p* < 0.001; R = −0.880, Figure 2C). Nevertheless, the logistic model revealed that this negative trend was not significant among male patients (*p* = 0.494, β = −0.014), only among females (*p* < 0.001, β = −0.073) and the total patient population (*p* = 0.003, β = −0.042).

The overall survival of patients was also analyzed. Among our melanoma patient group, the 5-year and 10-year overall survival rates were 78.3% and 68.9%, respectively. Women had significantly better overall survival compared to men (*p* < 0.001, Figure 2D). When patients were divided into two groups based on median age (=58 years), older patients (*p* < 0.0001; Figure 2E) showed relatively unfavorable outcomes. Breslow thickness also significantly impacted overall survival, since melanoma patients with a Breslow thickness below the median were associated with better outcomes (Figure 2F).

**Figure 3:**
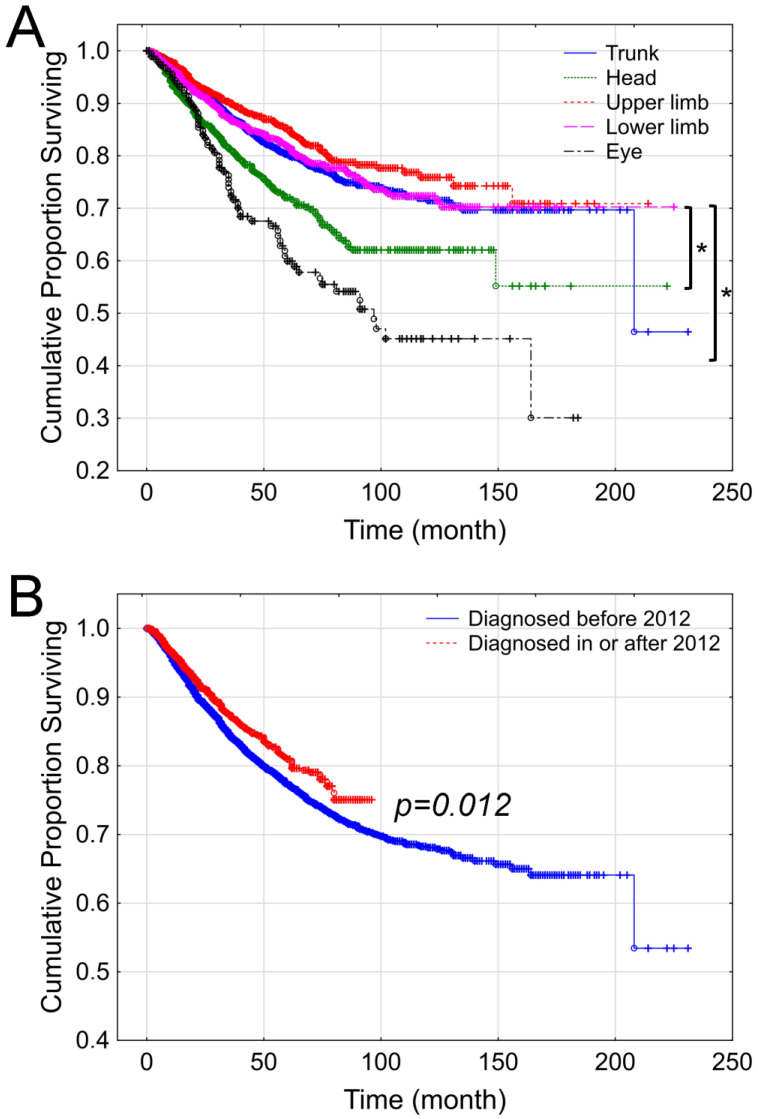
Impact of tumor localization (**A**) and date of diagnosis (**B**) on the overall survival of melanoma patients at the National Institute of Oncology between 2001 and 2018. (*: *p* < 0.001).

Lesions located on the trunk and upper extremities had better survival rates compared to melanomas on the head, neck, or eye (*p* < 0.001). In addition, melanomas on the lower extremities were associated with a better prognostic indicator compared to those on the head (*p* = 0.00006) and eye (*p* = 0.000001; Figure 3A).

Melanoma patient outcomes reflected the therapeutic advancements, with patients diagnosed after 2012 benefiting from improved survival due to the introduction of targeted therapy and immunotherapy (*p* = 0.012, Figure 3B).

For the analysis of different confounding factors on the overall survival of the studied melanoma patients, a Cox proportional hazard model was undertaken. Our analysis involved gender and age, Breslow depth, and localization of the primary tumor. Since in case of exulceration and histological subtype large pieces of data were missing, we excluded those parameters from the calculations. We also excluded those cases where data on the location of the primary tumor is relatively infrequent. The Cox model revealed that Breslow and age are the strongest independent predictors. Compared to simple log-rank statistics, tumor localization and gender proved to be insignificant (Table 3).

## 4. Discussion

According to GLOBOCAN estimates, in 2018, Hungary had the highest incidence and mortality rates for malignant tumors in Europe, with melanoma ranking as the 9th most common malignancy [11]. The incidence of melanoma in Hungary follows the steadily increasing European trends. On the other hand, other studies indicate stagnation over the past decade; in addition, in previous years, the potential overdiagnosis of melanoma has also been suggested [12]. However, information about prognostic factors is limited [10,13,14,15]. A growing number of studies are investigating correlations and changes between prognostic parameters and survival, helping to track melanoma characteristics. Our retrospective, single-center study provides insight into melanoma trends in Hungary over nearly two decades, which may allow for observations on the changing trends of prognosis. Examining our cohort, we found only minimal female predominance (53%). This aligns with findings from a previous study that examined a similar patient cohort treated for cutaneous melanoma at the National Institute of Oncology between 1998 and 2008, which also found no significant difference in incidence between the sexes [9,16]. The median age in our study was 58 years, closely matching the results of the previous study, indicating that patient data from the National Institute of Oncology has not changed significantly over the decades [16]. Our results also align with international trends, as melanoma is most commonly diagnosed between the ages of 40 and 60 worldwide, with a median age of 57 years [17]. However, the age-specific gender distribution observed worldwide is still a debated phenomenon. Several studies have attempted to explore the cause of higher incidence in young women, with findings that corroborate our results. Studies by Raimondi et al., Fears et al., and Buja et al. concluded that the female population is more likely to engage in activities associated with high sun exposure, including the use of tanning beds. Additionally, several other studies concluded that women generally adopt a more health-conscious lifestyle, seek medical care earlier for complaints, and are more attentive to skin health compared to men [18,19,20]. On the one hand, both our previous population-based analysis and the current study suggest that higher sun exposure and greater skincare awareness among women contribute to their higher incidence. On the other hand, male melanoma patients are typically diagnosed with a higher Breslow thickness, which is associated with less favorable survival outcomes in both our patient cohort and population-based analysis [9].

Breslow thickness, which measures the depth of tumor invasion, is considered the most important prognostic marker of primary melanoma [16]. Our study revealed a significant decrease in the median Breslow thickness at diagnosis over the years, especially among women, indicating earlier tumor detection. From these results, we inferred that the increase in melanoma incidence is driven by the detection of tumors at increasingly earlier stages with lower Breslow thickness. In 2001, the median Breslow value was 2.00 mm, whereas by 2018, it had decreased to 0.88 mm. This negative correlation is particularly pronounced in women, meaning that they are diagnosed with significantly lower Breslow thickness compared to men. This is attributed to the previously mentioned societal tendencies, suggesting that women are more likely to seek dermatological screenings earlier, leading to earlier melanoma detection [17,19].

The most common localization was the trunk, followed by the upper limb, lower limb, and the head and neck region. The distribution was similar in both sexes, with lower limb cases being more common than upper limb cases in women. According to a meta-analysis by Gandini et al., this difference may be attributed to different dressing and sunbathing habits between the sexes, as intermittent sun exposure and sunburns significantly increase the risk of developing melanoma [21]. Consistent with our results, in the literature data trunk localization was more common in men, while lower limb localization was more frequent in women [16]. This distribution was also observed in the previously mentioned Italian study by Buja et al., where trunk localization was predominant in men (59.3%) and lower limb localization was most common in women (32.1%), with their percentages closely matching our results [22]. Similarly, Önnefält et al. analyzed data from 68,666 Swedish patients diagnosed with cutaneous malignant melanoma between 2004 and 2018, finding that trunk localization was the most frequent in men (51%). Among women, lower limb localization was the most common in 2004 (33%), but by 2018, trunk localization had become dominant in this group as well [20].

Melanomas on the head were discovered with significantly higher Breslow values compared to those on the trunk and upper limb regions. According to a study by Saaiq et al., this is likely because flat lesions on the scalp are much harder to detect, leading to delayed diagnosis. As a result, malignant melanomas in this region are often only noticed at a more advanced stage, when they may have already ulcerated or become elevated [23]. In a retrospective study conducted between 2008 and 2014, Lovasik et al. found that in almost 10% of referred head and neck melanoma cases, the tumor was first detected by the patient’s hairdresser, highlighting the significance of the beauty industry in identifying hard-to-diagnose localizations [24]. Beyond the difficulty of performing self-examination in the head–neck region, our results suggest that this issue is particularly pronounced in older men. In this population, melanomas were discovered with significantly higher Breslow values compared to those on the trunk or upper limb regions [25].

A population-level Hungarian mortality analysis was performed on 15,502 patients who died due to malignant melanoma between 2001 and 2019. Consistent with our findings, the study revealed that men are more likely to be victims of melanoma than women [9]. When examining survival, it is clear that Breslow thickness remains the most reliable prognostic indicator. Consequently, women have better survival outcomes than men, as their median Breslow value has decreased more steeply over the years. Age is also a significant prognostic factor, with patients under 58 years of age having a higher survival rate. This aligns with the observation that tumors tend to be detected at lower Breslow values in younger individuals [15]. In terms of localization, patients with melanoma on the trunk and upper or lower limbs had significantly better survival rates compared to those with head or uveal involvement. This is due to the late-stage diagnosis and higher Breslow values of melanomas in the head and neck region, which are harder to detect, as previously discussed [23,24]. Larsen et al. described the particularly poor prognosis of 139 Danish patients treated for conjunctival melanoma between 1960 and 2012, especially for tumors affecting the extrabulbar conjunctiva and neighboring tissue structures, due to their early metastatic potential [26].

In the early 2010s, revolutionary therapeutic options, including targeted therapies and immunotherapies, were introduced for advanced, metastasizing tumors. Between 1986 and 2013, the overall mortality rate among white populations in the US increased by 7.5%. However, since 2011, the US Food and Drug Administration (FDA) has approved 10 new treatments for metastatic melanoma, resulting in a 17.9% increase in overall survival from 2013 to 2016 [27]. Our single-center study results also show that survival was more favorable for tumors discovered after the introduction of novel treatment modalities. This result is in accordance with two previously published Hungarian studies that applied population-level data [9,10]. One indicator of the quality of cancer treatment is the mortality and incidence rate (MIR). According to a retrospective study from 2021, the MIR in Hungary decreased by about 0.035 between 2011 and 2018 [28]. However, our study has limitations, primarily its retrospective design, which raises concerns about data reliability.

On the other hand, our work contained some limitations. In contrast to the outstanding sample size, some parameters were not available in a significant portion of the cases. For instance, Breslow Depth was present in 80.6%, and exulceration of the primary lesion only in 28.5% of the cases. Changes in the diagnostic process over time were also ignored. Nevertheless, in contrast to the limited data, we think that our work may add new details to the big picture.

This study has important public health implications; despite the increasing incidence of melanoma in Hungary, the median Breslow thickness values and consequently mortality data are improving. This supports the critical role of secondary prevention and its effectiveness, as well as the need for the continued development of novel therapeutic options [9,16].

## 5. Conclusions

Based on our single-center clinical sample, we concluded that despite the rising incidence of melanoma in Hungary, the annual median Breslow thickness showed a decreasing trend, accompanied by improved survival. This highlights the critical role of secondary prevention measures and their effectiveness, as well as the impact of novel therapeutic advancements.

## Figures and Tables

**Table 1:** General characteristics of patient group treated for melanoma at the National Institute of Oncology, Hungary, between 2001 and 2018.

Total Number of Patients (N; %)	6267 (100%)
Age (years) (median, range)	58 (7–94)
Gender	
Male	2970 (47.4%)
Female	3297 (52.6%)
Breslow (mm) (median, range)	1.22 (0.03–50)
Follow-up (months) (median, range)	38 (0–231)
Status (N; %)	
Alive	5122 (81.7%)
Dead	1145 (18.3%)
Location (N; %)	
Trunk	2776 (45.3%)
Head and neck region	690 (11.3%)
Upper extremities	1061 (17.3%)
Lower extremities	1320 (21.5%)
Uveal	194 (3.2%)
Occult	44 (0.7%)
Mucosal	11 (0.2%)
Genital region	25 (0.4%)
Anal region	10 (0.2%)
Histological subtype (N; %)	
Superficial spreading	2216 (35.4%)
Nodular	798 (12.7%)
Lentigo melanoma maligna	152 (2.4%)
Ocular	193 (3.1%)
Mucosal	33 (0.5%)
Other	24 (0.4%)
No data available	2800 (44.7%)
Exulceration of primary (N; %)	
No	1183 (18.8%)
Yes	603 (9.6%)
No data available	4481 (71.5%)
Clark level (N; %)	
I	455 (7.3%)
II	1044 (16.7%)
III	1829 (29.2%)
IV	1901 (30.3%)
V	314 (5%)
No data available	724 (11.6%)

**Table 2:** Comparison of melanoma patients treated at the National Institute of Oncology, Hungary, between 2001 and 2018, by gender.

	Male	Female	*p*
Total number of patients	2970 (47.4%)	3297 (52.6%)	
Age (in years) (median; range)	60.5 (7–94)	55 (10–93)	*p* < 0.001
Breslow (mm) (median; range)	1.5 (0.05–45)	1.075 (0.03–50)	*p* < 0.001
Localization (N, %)			*p* < 0.001
Trunk	1574 (54.4%)	1202 (37.1%)	
Head and neck region	383 (13.2%)	307 (9.5%)	
Upper extremities	474 (16.4%)	587 (18.1%)	
Lower extremities	348 (12.2%)	971 (30.0%)	
Eye	79 (2.7%)	115 (3.6%)	
Occult	25 (0.9%)	19 (0.6%)	
Mucosa	4 (0.1%)	7 (0.2%)	
Genital region	2 (0.1%)	23 (0.7%)	
Anal region	5 (0.2%)	5 (0.2%)	
Exulceration (N; %)			*p* < 0.001
No	529 (17.8%)	654 (19.8%)	
Yes	328 (11.0%)	275 (8.3%)	
No data available	2113 (71.1%)	2368 (71.8%)	

**Table 3:** Multivariate Cox regression model for the main clinicopathological factors on the overall survival of melanoma patients at the National Institute of Oncology between 2001 and 2018.

	RR (95% CI)	*p*
Age	1.013 (1.005–1.022)	0.002
Gender (female—reference: male)	0.787 (0.606–1.022)	0.073
Breslow	1.217 (1.21–1.225)	<0.001
Location (lower extremities—reference: upper extremities)	1.142 (0822–1.586)	0.912
(head and neck—reference: upper extremities)	1.27 (0.794–2.032)	0.462
(trunk—reference: upper extremities)	1.103 (0.785–1.549)	0.906

## Data Availability

Upon special request, the authors will share anonymized datasets.
